# Resonator-Inspired Metamaterial Sensor: Design and Experimental Validation for Measuring Thickness of Multi-Layered Structures

**DOI:** 10.3390/s18124213

**Published:** 2018-12-01

**Authors:** Mohammad Tariqul Islam, Md. Naimur Rahman, Md. Samsuzzaman, Mohd Fais Mansor, Norbahiah Misran

**Affiliations:** 1 Centre of Advanced Electronic and Communication Engineering, Universiti Kebangsaan Malaysia, UKM Bangi, Selangor 43600, Malaysia; samsuzzaman@ukm.edu.my (M.S.); m.mansor@ukm.edu.my (M.F.M.); bahiah@ukm.edu.my (N.M.); 2 Patuakhali Science and Technology University, Patuakhali 8602, Bangladesh

**Keywords:** metamaterial sensor, resonator, thickness, sensitivity

## Abstract

A digit 8-shaped resonator inspired metamaterial is proposed herein for sensor applications. The resonator is surrounded by a ground frame and excited by a microstrip feedline. The measurement of the sensor can be performed using common laboratory facilities in lieu of using the waveguide, as the resonator, ground frame, and feedline are all on the same microstrip. To achieve metamaterial properties, more than one unit cell is usually utilized, whereas, in this work, a single cell was used to achieve the metamaterial characteristics. The properties of the metamaterial were investigated to find the relationship between the simulation and measurements. The proposed metamaterial sensor shows considerable sensitivity in sensor application. For the sensor application, FR4 and Rogers RO4350 materials were used as the over-layer. The sensor can measure dielectric thickness with a sensitivity of 625 MHz/mm, 468 MHz/mm, and 354 MHz/mm for the single over-layer, double over-layers, and multiple over-layers, respectively. The proposed prototype can be utilized in several applications where metamaterial characteristics are required.

## 1. Introduction

Since their first introduction in 1968, metamaterials attracted researchers working with the characterization of metamaterials. Researchers introduced several resonator-based metamaterials which work in different frequencies [1]. For ultra-wideband microwave imaging, a split-ring resonator (SRR)-inspired metamaterial antenna was utilized [2]. Terahertz sensing was described using an SRR-based metamaterial [3]. Again, a terahertz-sensing device was introduced in Reference [4] using a broadside-coupled triangular SRR-based metamaterial. A microwave metamaterial sensor was introduced where transmission lines worked as the building block [5]. A complementary SRR was used to characterize the dielectric properties of a metamaterial operating in the 0.8–1.3 GHz band [6]. Dielectric characterization of the microfluidics was done with a metamaterial sensor using a microstrip-coupled complementary split-ring resonator (CSRR) [7]. An H-shaped resonator-based metamaterial was developed and experimented in sensor and absorber applications [8]. To find out the resonant frequencies and Q-factors of whispering-gallery modes (WGMs), a microring resonator-based metamaterial sensor was introduced [9]. For the calculation of resonant frequencies and the electric field distribution of WGMs, a negative permeability metamaterial sensor was developed and described in Reference [10]. A metamaterial-based wireless sensor was used to measure strain and displacement during the deformation of elastic and plastic materials, which is beneficial in health-monitoring systems [11]. An S-shaped split-ring resonator (SSRR)-based metamaterial sensor, using a coplanar waveguide (CPW) transmission line, was used to find the angular velocity and displacement [12]. An SRR-inspired metamaterial-based microfluidic sensor was utilized to identify, classify, and characterize biochemical and chemical analyses [13]. A CSRR-based sensor was used to measure the thickness and permittivity of a multi-layered structure using the non-invasive microwave method [14]. A quasi-static resonator was used to measure the non-destructive thickness, which also used a CSRR operating between 1.60 to 2.30 GHz bands [15]. A planar resonator sensor was utilized to monitor the solid element in a lossy medium operating at 1.50 GHz having a quality factor of 230 [16]. A metamaterial-based microwave sensor was introduced to measure the impurities in a liquid based on the transmission quality of the sensor [17]. A resonator-based microwave sensor was used to monitor the sensitivity enhancement for CO_2_ detection [18]. A differential sensor based on two SRRs was utilized to characterize solid dielectric samples [19]. A microwave gas sensor was represented using electromagnetic transducers to monitor volatile organic particles [20]. A wireless biosensor was rendered for the detection of bacteria in milk using a capacitor-inductance tank [21]. A microstrip gas sensor based on a ring resonator was introduced, and was used to detect humidity and ammonia [22]. A substrate-integrated waveguide sensor was presented as a chemical sensor, using ethanol and deionized water as dielectric fluids [23]. Most of the reviewed sensors are large in size and complex to design. Again, some sensors stated above have limited sensitivity.

In this article, a resonator-inspired metamaterial sensor is proposed based on microstrip technology. Simulated and experimented metamaterial properties were investigated extensively, and both results matched quite well. Finally, the metamaterial sensor was utilized to identify unknown materials of different thickness. The proposed sensor has better sensitivity than the earlier reported similar works.

## 2. Model, Prototype, and Experiment

**Figure 1:**
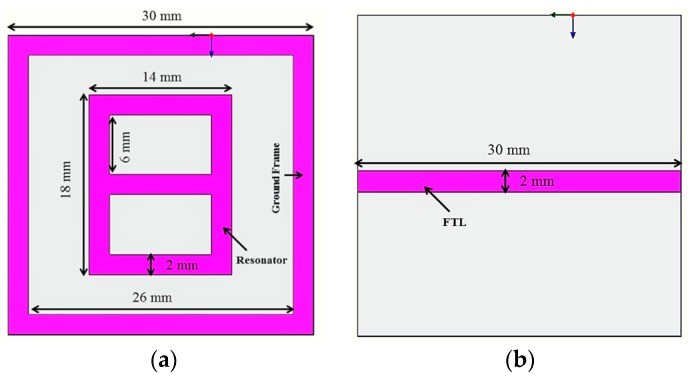
Geometry of the sensor: (**a**) top side, and (**b**) bottom side.

**Figure 2:**
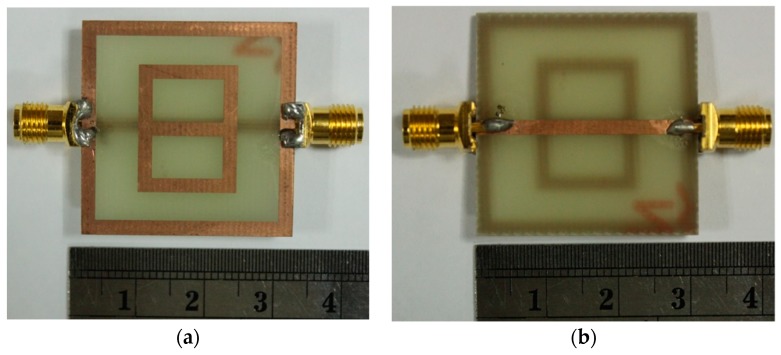
A prototype of the sensor: (**a**) top side, and (**b**) bottom side.

**Figure 3:**
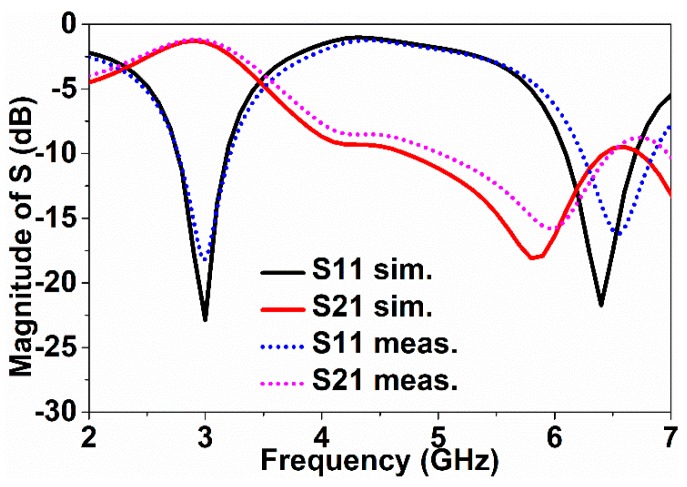
The magnitude of the S-parameter.

The metamaterial consists of a digit 8-shaped resonator, surrounded by a ground frame, along with a feeding transmission line (FTL) exhibiting metamaterial characteristics. The metamaterial was prototyped on the FR4 material, which has 4.6 relative permittivity and 0.02 loss tangent. The resonator and the ground frame were sketched on the same portion of the substrate. The feeding transmission line (FTL) was sketched on the opposite portion of the substrate. The excitation is supplied to the resonator through the FTL. The measurement of the sensor can be performed using common laboratory facilities in lieu of using the waveguide, as the resonator, ground frame, and feedline are all on the same microstrip. To achieve metamaterial properties, more than one unit cell is usually utilized, whereas, in this work, a single cell was used to achieve the metamaterial characteristics. The design of the resonator is simpler compared to the previously reported planar resonator-based sensors. Again, the previously reported similar works used larger resonator sensors to detect the over-layers. Moreover, some sensors have limited sensitivity as compared to the proposed sensor. The metamaterial was designed and investigated using the High-Frequency Structural Simulator (HFSS) software. The geometry of the sensor is shown in Figure 1. Figure 2 depicts the prototype of the digit 8-shaped resonator-inspired metamaterial sensor. The N5227A PNA Microwave Network Analyzer (Agilent Technologies, Santa Clara, CA, USA) (10 MHz–67 GHz) has been used to measure the sensor. The reflection and transmission coefficients of the sensor are depicted in Figure 3, where resonances were found at 3 GHz and 6.5 GHz. The transmission resonant frequency was observed at 6.0 GHz.

## 3. Characterization of Metamaterial

The Nicolson-Ross-Weir (NRW) method was used to characterize the metamaterial properties. The values of the refractive index depend on the values of the permeability and permittivity. The NRW method is mathematically expressed by the subsequent equations [1].

(1) $$\Gamma =\left[\left(S_{11}^{2}+S_{21}^{2}+1\right)2S_{11}\right]\pm \sqrt{{\left[\left(S_{11}^{2}+S_{21}^{2}+1\right)2S_{11}\right]}^{2}-1},$$

(2) $$T=\frac{\left(S_{11}+S_{21}-\Gamma \right)}{\left[1-\Gamma \left(S_{11}+S_{21}\right)\right]},$$

(3) $$\kappa =\left[\log\left(\frac{1}{\left|T\right|}\right)\right]+j\left[2m\pi -phase\left(T\right)\right]/d,\;m=\;0,\;\pm 1,\;\pm 2,\;\pm 3,$$

(4) $$\varepsilon =\frac{\kappa }{\kappa_{0}}\;\left(\frac{1-\Gamma }{1+\Gamma }\right),$$

(5) $$\mu =\frac{\kappa }{\kappa_{0}}\;\left(\frac{1+\Gamma }{1-\Gamma }\right),$$

where *μ* denotes permeability, ε denotes permittivity, and $\kappa_{0}$ denotes the propagation constant in free space. Using these values of permeability and permittivity, the effective constituents were calculated, following the Drude model dispersion.

**Figure 4:**
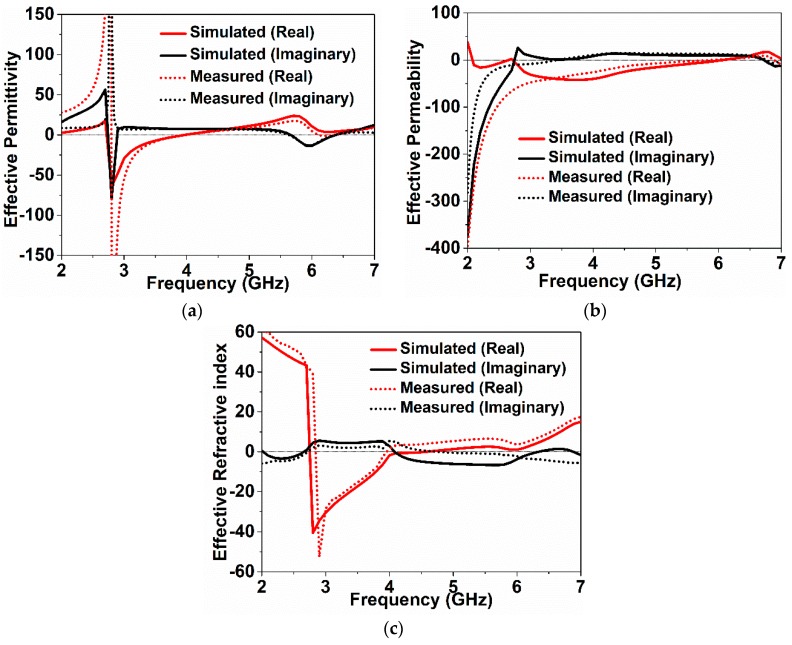
Effective parameters of the sensor: (**a**) permittivity, (**b**) permeability, and (**c**) refractive index.

At the peak of the transmission (*S*_21_), the effective permittivity and permeability became negative. In Figure 4a, the effective permittivity remains negative from 2.7 GHz to 4.1 GHz, whereas Figure 4b depicts a negative permeability from 2 GHz to 6 GHz. The refractive index became negative as the permittivity and permeability remained negative, as shown in Figure 4c. Therefore, the metamaterial depicted double-negative properties in the bandwidth from 2.7 GHz to 4.1 GHz, and all parameters had a negative peak at 3 GHz [1,24].

**Figure 5:**
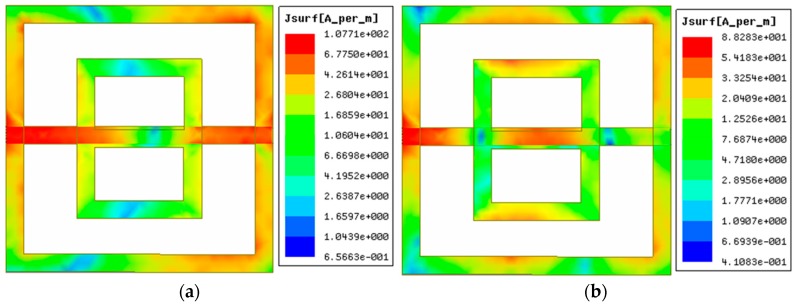
Current distribution of the sensor: (**a**) at 3.0 GHz, and (**b**) at 6.5 GHz.

To describe the resonance type, the surface current distribution of the sensor was included. Figure 5 represents the surface current distribution of the sensor.

**Figure 6:**
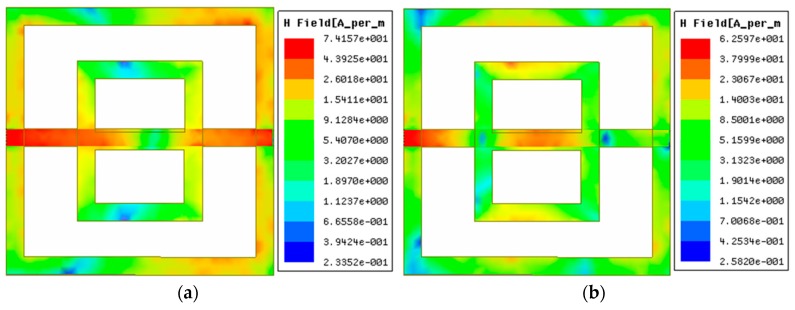
Magnetic field distribution of the sensor: (**a**) at 3.0 GHz, and (**b**) at 6.5 GHz.

The current was distributed mainly on the resonator and the FTL. Two opposite directional magnetic dipoles were formed on the ground frame due to the symmetrical rotation of the surface currents on the resonator. Figure 6 represents the magnetic field distribution of the sensor.

The FTL was mutually attached to the ground and the resonator with its electric field. Hence, the sensor presents inductive and capacitive behavior because of the ground frame and the gap between the ground and the resonator. Diamagnetism appears due to the induced magnetic field, which is opposite to the external magnetic field. For the diamagnetism, the sensor shows negative permeability. Moreover, the ground frame creates negative permittivity and negative permeability through the inductive-capacitive properties of the sensor.

## 4. Metamaterial as Sensor

The resonator-inspired metamaterial sensor was used in a double-sided sensor application. Unknown objects were detected, upon being used as the over-layer. The sensor was experimented using different over-layers with different thickness by means of the change in frequency responses. The sensitivity of the sensor was determined considering the change in dielectric thickness with the permittivity kept constant.

**Figure 7:**
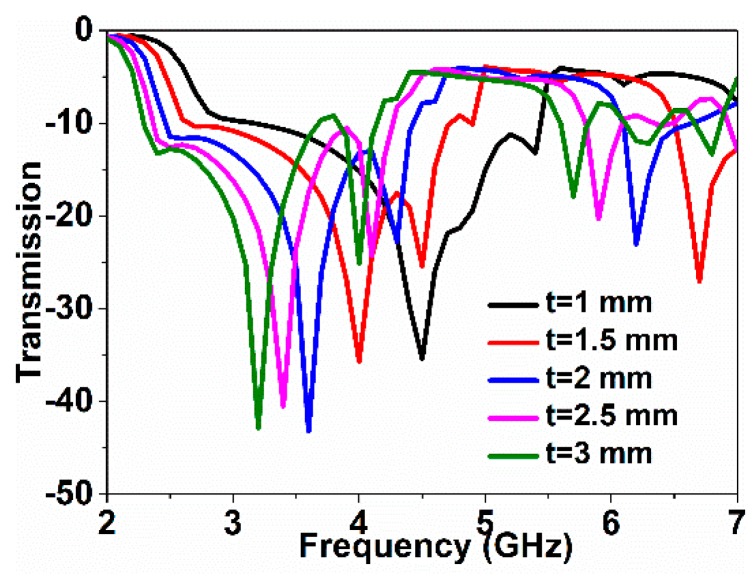
Change in transmission with the change in over-layer thickness.

The frequency responses for changes in over-layer thickness were observed. In the simulation, an over-layer having 4.4 dielectric constants was used, as it has parameter values close to the FR4 substrate. The simulated frequency responses with the changes in over-layer thickness are shown in Figure 7. The resonance shifted downward when over-layer thickness increased. The metamaterial sensor with the over-layer constitutes a resistor/inductor/capacitor (RLC) circuit, where the resonance is determined by effective capacitance and inductance. As effective inductance and capacitance vary when the over-layer thickness increases due to the self-resonance, the resonance shifts downward because of the resonance frequency changes with the effective capacitance and inductance. As the ground plane around the resonator provides additional capacitance and inductance, it has significant effects on the resonance frequency.

**Figure 8:**
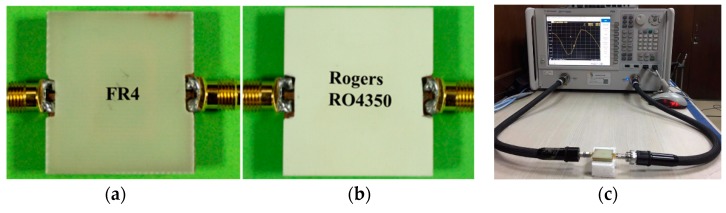
Sensors with over-layers: (**a**) FR4 over-layer, and (**b**) Rogers RO4350 over-layer. (**c**) PNA measurement set-up.

**Figure 9:**
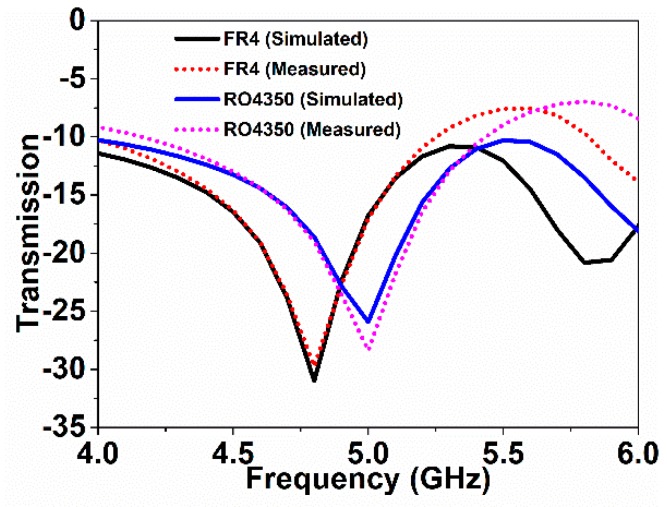
Transmission responses with FR4 and Rogers RO4350 over-layers.

In the measurement, the FR4 substrate, with 1.66 mm thickness with 4.6 relative permittivity, and the Rogers RO4350 substrate, with 1.53 mm thickness with 3.66 relative permittivity, were utilized as the over-layers. Figure 8 depicts the sensor with over-layers along with the PNA measurement set-up. Figure 9 depicts the simulated and experimental resonance responses of the metamaterial sensor when detecting the unknown material.

**Figure 10:**
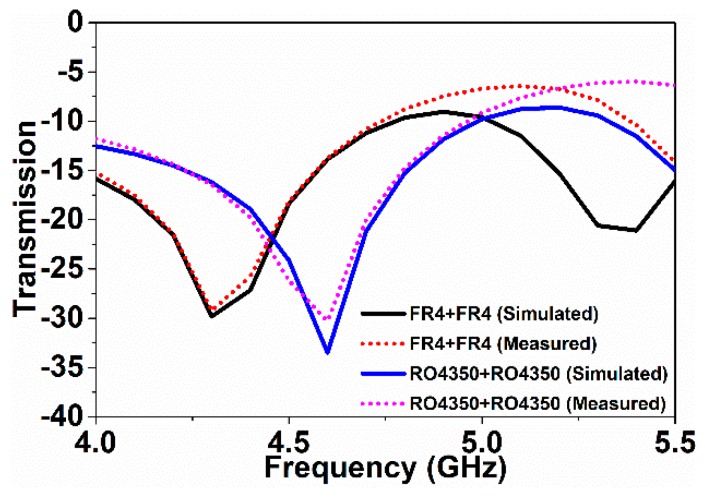
Transmission variances with FR4 and Rogers RO4350 double over-layers.

The frequency shifts depend on the over-layer thickness and the over-layer’s permittivity. The larger the values of thickness and permittivity are, the more the resonance shifts downward. The higher the over-layer thickness and permittivity become, the larger the downshift of the resonance becomes. In this article, the sensitivity of the sensor was measured considering the change in over-layer thickness upon keeping permittivity constant. In Figure 9, the resonance moved downward when the over-layer thickness increased. The over-layer thickness was increased by adding etched substrates, making a stack of substrates. There existed negligible gaps between the stack of substrates. However, the measured results differed in some cases due to the little gaps between the stacked substrates. The thickness of FR4 is larger than that of the Rogers RO4350 substrate; thus, for the FR4 over-layer, the resonance decreases further than the Rogers RO4350 over-layer. For specific clarification, the transmission resonance was at 6.0 GHz with no over-layer; however, using the Rogers RO4350 and FR4 over-layers, the resonances moved to 5.0 GHz and 4.8 GHz, respectively. As the thickness increased, the resonance frequency shifted downward. The greater the thickness of the over-layer became, the more the resonance shifts occurred. Using the shifting values of the resonance frequency, the sensitivity of the sensor was calculated. Considering the frequency shift as a function of over-layer thickness, the sensitivity of the metamaterial sensor was 625 MHz/mm for a single over-layer. For a double over-layer, FR4 + FR4 and Rogers RO4350 + Rogers RO4350 substrates were utilized. Figure 10 depicts the resonance responses for the double over-layers.

The resonance moved downward when the thickness of the over-layer increased. Hence, for the Rogers RO4350 + Rogers RO4350 and FR4 + FR4 double over-layers, the resonances moved to 4.6 GHz and 4.3 GHz, respectively. Considering the frequency shift as a function of over-layer thickness, the sensitivity of the metamaterial sensor was 468 MHz/mm for the double over-layer.

**Figure 11:**
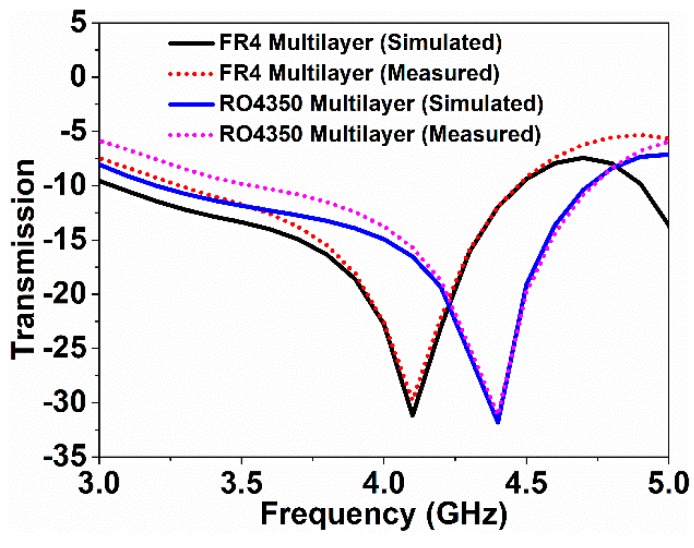
Transmission variances with FR4 and Rogers RO4350 multiple over-layers.

For a further extension of the experiment, multiple over-layers were considered. In this case, FR4 + FR4 + FR4 and Rogers RO4350 + Rogers RO4350 + Rogers RO4350 substrates were utilized. The simulated and experimented results are plotted in Figure 11. The resonance downshifted when the thickness increased. Using the Rogers RO4350 + Rogers RO4350 + Rogers RO4350 and FR4 + FR4 + FR4 multiple over-layers, the resonances downshifted to 4.4 GHz and 4.1 GHz, respectively. Considering the frequency shift as a function of over-layer thickness, the sensitivity of the metamaterial sensor was 354 MHz/mm for the multiple over-layers experimented above.

For the over-layer, double over-layer, and multiple over-layer variants, the simulated and measured values agreed well. On the basis of the simulations and measurements, it can be concluded that the metamaterial sensor can measure the dielectric thickness of multi-layered structures with considerable sensitivity.

The proposed sensor outperformed previous related works according to sensor size, sensor type, and sensitivity. Table 1 shows the comparative characteristics between the proposed sensor and the previous related sensors.

## 5. Conclusions

A digit-8 shaped resonator-inspired metamaterial sensor was modeled, prototyped, and analyzed herein. The sensor was experimented using usual laboratory apparatus in lieu of waveguides. Both the simulated and measured values matched well, showing the double-negative metamaterial characteristics of the metamaterial sensor in the operating region. The proposed sensor measured the over-layer thickness, using FR4 and Rogers RO4350 substrates as the over-layers. The sensor was used to measure the dielectric thickness of multi-layered structures with considerable sensitivities of 625 MHz/mm, 468 MHz/mm, and 354 MHz/mm for the single over-layer, double over-layers, and multiple over-layers, respectively.

## Figures and Tables

**Table 1:** Comparative characteristics of the proposed sensor with previous related sensors. SRR—split-ring resonator; SSRR—S-shaped SSR; TSRR—triangular SRR; CSRR—complementary SRR.

Reference	Sensor Size	Resonator	Operating Band	Sensing Base	Sensitivity
[1]	35 mm × 35 mm	S-shaped	GHz	Frequency shift	Not reported
[3]	87.5 µm × 87.5 µm	SSRR	THz	Frequency shift	Not reported
[4]	75 µm × 75 µm	TSRR	THz	Frequency shift	Not reported
[7]	Not reported	CSRR	GHz	Frequency shift	400 MHz
[19]	Not reported	SRR	GHz	Frequency shift	72 MHz
Proposed	30 mm × 30 mm	8-shaped	GHz	Frequency shift	Max 625 MHz
